# Supplementary material for: Traveling salesman problem solution using magnonic combinatorial device
Source: Sci Rep. 2023 Jul 20;13:11708. doi: 10.1038/s41598-023-38839-7 (PMC10359328; doi:10.1038/s41598-023-38839-7)
Supplement: Supplementary file 1 — Supplementary Information. [file 41598_2023_38839_MOESM1_ESM.docx]

Supplementary Materials for

**Traveling Salesman Problem solution using Magnonic Combinatorial Device**

| **Cities** | **Distances [miles]** |
| --- | --- |
| Los Angeles – Miami | 2342 |
| Los Angeles – Chicago | 1741 |
| Los Angeles – Washington | 2288 |
| Los Angeles – Las Vegas | 236 |
| Los Angeles – San Francisco | 347 |
| Miami – Chicago | 1189 |
| Miami – Washington | 920 |
| Miami – Las Vegas | 2171 |
| Miami – San Francisco | 2590 |
| Chicago – Washington | 594 |
| Chicago – Las Vegas | 1039 |
| Chicago – San Francisco | 1853 |
| Washington – Las Vegas | 2085 |
| Washington – San Francisco | 2435 |
| Las Vegas – San Francisco | 416 |

Table [s1] with city-to-city distances used in numerical simulations. The distances between the cities are taken from the Google Map application.


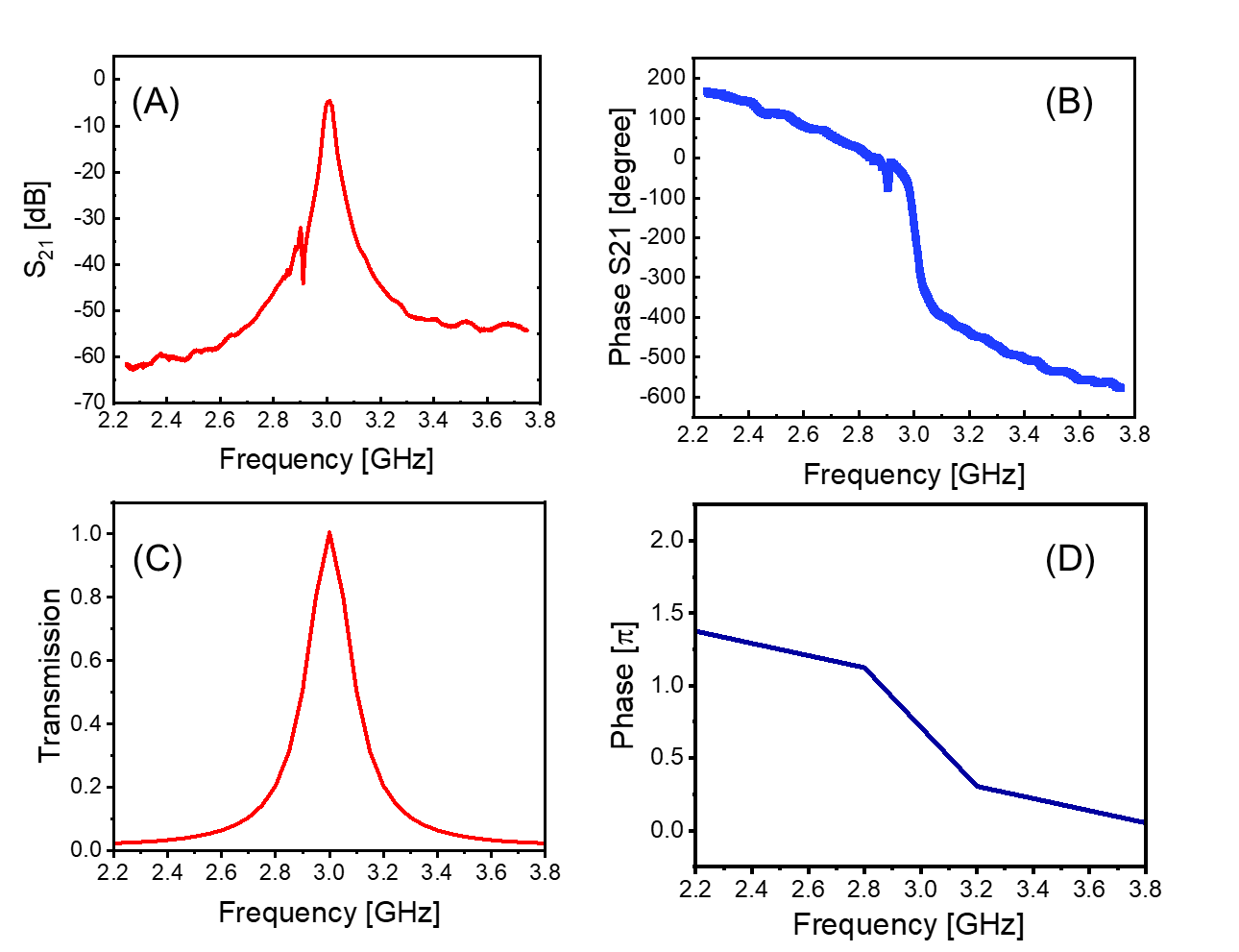


Figure [s1]: YIG-based frequency filter produced by Micro Lambda Wireless, Inc, model MLFD-40540. (A) Experimental data: S21 parameter (amplitude) of the commercial filter. (B) Experimental data: S21 parameter (phase shift) of the commercial filter. (C) Results of numerical fitting: transmission of the spin wave element. (D) Results of numerical fitting: phase shift produced by the spin wave element. The data are shown in the frequency range from 2.2 GHz to 3.8 GHz.


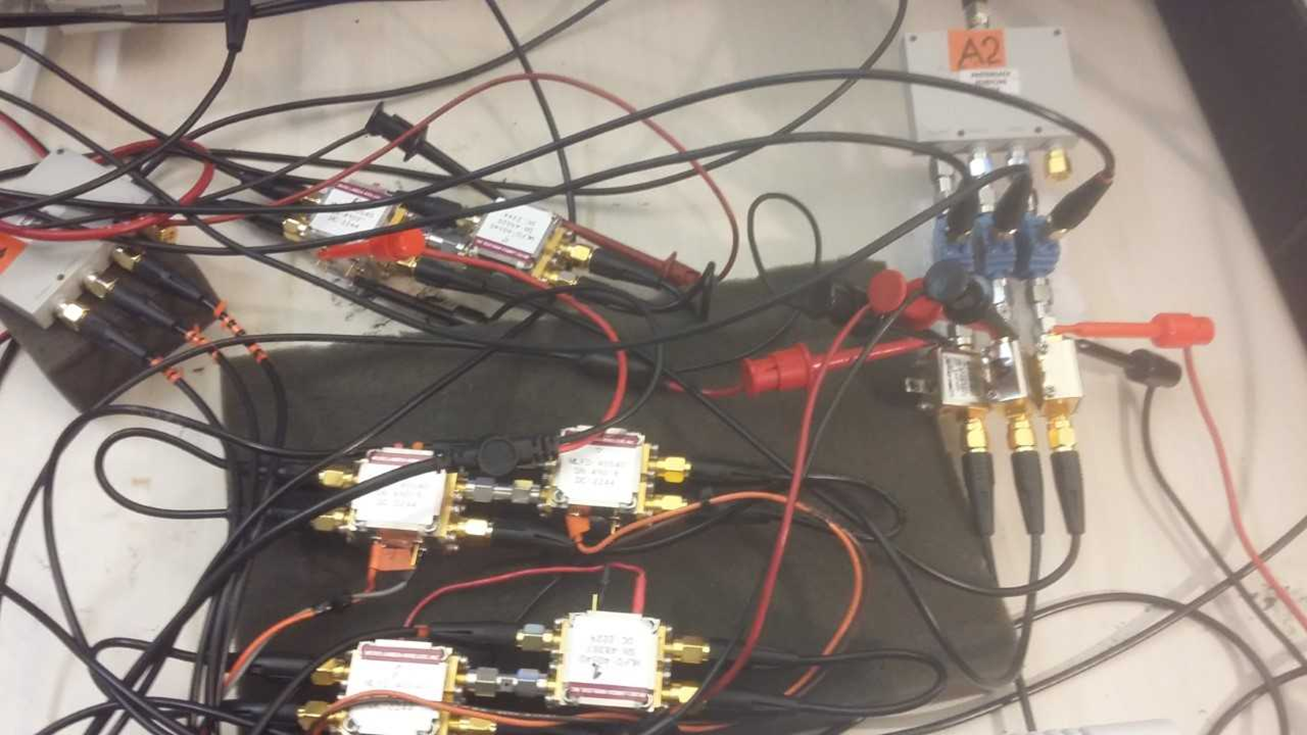


Figure [s2]: Photo of the prototype device. The prototype is built of YIG-based frequency filters produced by Micro Lambda Wireless, Inc, model MLFD-40540 connected by coaxial cables through the set of splitters and combiners.


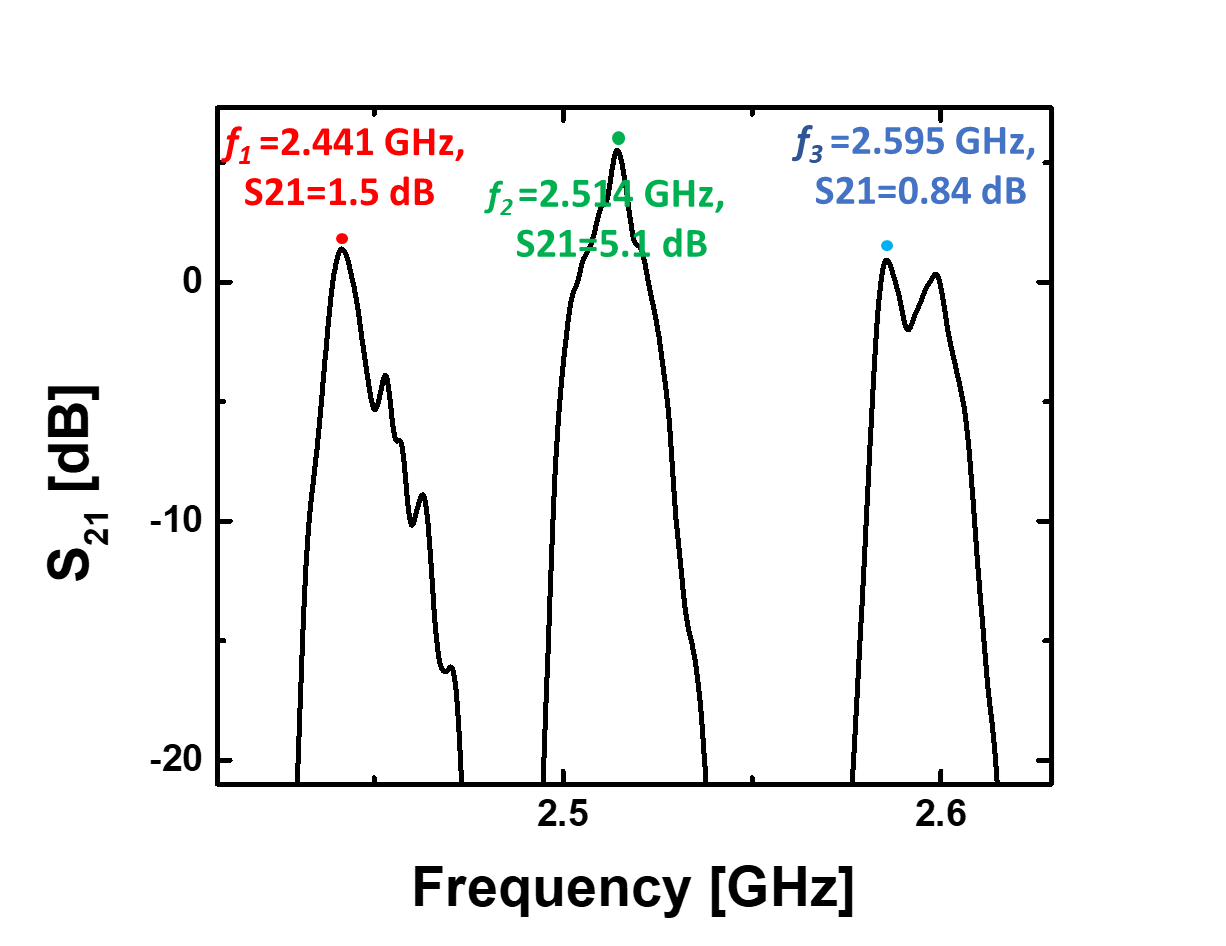


Figure [s3]: Experimental data: Transmission of the mesh for the three routes. Data are taken with VNA. The mesh is not connected to the electric part (i.e., no amplification)


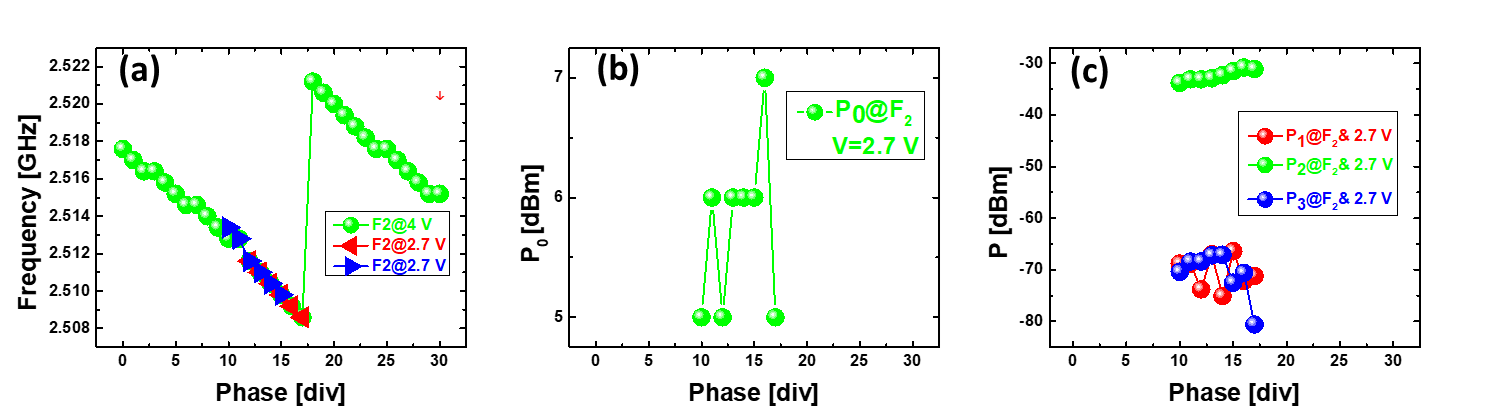


Figure [s4]: Experimental data: Auto-Oscillations in the Active Ring Circuit. Magnetic mesh is connected to the electric part. Auto-oscillations are observed for certain levels of amplification and the external phase shifter. The gain in the circuit is set to +13.5 dB.

(a) Frequencies of the auto-oscillations depending on the external phase shifter. One div is equivalent to π/30 radians (b) Total power in the active ring circuit depending on the external phase. (c) Power of the auto-oscillations in different propagation routes.
